# Prenatal 3.5 GHz radiofrequency exposure induces renal histological changes and DNA damage in 6-month-old rats

**DOI:** 10.1007/s00418-026-02504-7

**Published:** 2026-07-09

**Authors:** Elif Gelenli Dolanbay, Fazile Canturk Tan, Hava Bektas, Sumeyye Koc, Salih Varol, Omer Kilic, Unal Uslu, Suleyman Dasdag

**Affiliations:** 1 Department of Histology and Embryology, Faculty of Medicine, Istanbul Medeniyet University, Unalan Neighborhood, Unalan Street, D-100 Highway Side Road Uskudar, 34700 Istanbul, Turkey; 2 Department of Biophysics, Faculty of Medicine, Erciyes University, Kayseri, Turkey; 3 Department of Biophysics, Faculty of Medicine, Van Yuzuncu Yil University, Van, Turkey; 4 Department of Molecular Biology and Genetics, Bogazici University, Istanbul, Turkey; 5 Department of Biophysics, Faculty of Medicine, İstanbul Medeniyet University, Istanbul, Turkey

**Keywords:** 3.5 GHz, Radiofrequency radiation, Kidney, Autophagy, DNA damage, Rat

## Abstract

Early-life environmental stressors may influence long-term organ development and cellular homeostasis. This study investigated whether prenatal exposure to 3.5 GHz radiofrequency radiation (RFR) is associated with renal structural alterations, autophagy-related changes, and DNA damage in adult rat offspring. A total of 24 pregnant Wistar Hannover rats were randomly assigned to sham control, exposure during the last 2 weeks of gestation (D2T: exposure during the last 2 weeks of gestation), or exposure throughout gestation (D3T: exposure throughout gestation) groups (*n* = 8 dams per group). Male offspring were selected and euthanized at 6 months of age. Kidney tissues were examined histopathologically for structural alterations. Autophagy-related markers (Beclin-1 and LC3) were assessed by immunohistochemistry, and DNA damage was evaluated using the comet assay. Statistical analyses were performed using one-way analysis of variance (ANOVA) followed by appropriate post hoc tests. Peak spatial specific absorption rate (psSAR) values in the uterine region were 0.06622 mW/g (1 g averaging) and 0.03825 mW/g (10 g averaging). Prenatal RFR exposure was associated with significant renal histopathological alterations in offspring, most pronounced in the D3T group, including glomerular atrophy, tubular dilation, epithelial vacuolization, and cast formation (*p* < 0.01–0.001 versus controls). Beclin-1 and LC3 expression levels were significantly increased in both exposure groups (*p* < 0.001), suggesting altered autophagy-related marker expression. The comet assay demonstrated significantly increased DNA fragmentation in D2T and D3T groups compared with controls (*p* < 0.001), indicating increased genomic stress. Overall, prenatal exposure to 3.5 GHz RFR is associated with renal structural alterations, increased DNA damage, and changes in autophagy-related markers in adult rat offspring.

## Introduction

With the widespread use of wireless communication technologies, concerns regarding the potential health risks of RFR exposure have gained increasing attention. The kidneys, essential organs for maintaining homeostasis, are particularly vulnerable to environmental stressors, including electromagnetic fields (EMFs) (Mahmoudi et al. 2018; Hasan et al. 2021; Zhang et al. 2023). Studies have suggested that prolonged RFR exposure may be associated with cellular stress responses, including processes reported in the literature such as oxidative stress (Oktem et al. 2005; M Fahmy and Mohammed 2015; Berkoz et al. 2018), inflammation (Bodera et al. 2015; Khattab and Marei 2019), and cellular damage in renal tissues (Chauhan et al. 2017; Hasan et al. 2021), potentially contributing to impaired kidney function. However, most existing research has focused on lower-frequency RFR (e.g., 900 MHz, 1800 MHz, and 2.45 GHz Wi-Fi), leaving the biological effects of higher-frequency RFR, such as 3.5 GHz, largely unexplored.

Previous research indicates that RFR exposure has been proposed to be associated with alterations in redox homeostasis, including changes in reactive oxygen species (ROS) levels and antioxidant defense mechanisms, with possible implications for lipid peroxidation, protein oxidation, and mitochondrial function (Ratliff et al. 2016; Dasdag and Akdag 2016). Additionally, chronic RFR exposure has been linked to DNA damage (Verschaeve et al. 2010; Akdag et al. 2018; Bektas et al. 2020; Kaur et al. 2023) and dysregulated autophagic processes (Jangid et al. 2024; Kirimlioglu et al. 2024), both of which are critical for maintaining cellular homeostasis and tissue repair. Despite these findings, the extent to which higher-frequency RFR affects these mechanisms in renal tissues remains unclear.

Emerging evidence suggests that prenatal RFR exposure may pose an even greater risk, as the developing kidney is particularly susceptible to environmental stressors during gestation. Experimental studies indicate that prenatal EMF exposure can lead to structural and functional alterations in renal development (Kilic et al. 2023; Pawar and Martin 2024), including glomerular damage, tubular degeneration, and cellular dysfunction potentially involving processes reported in the literature such as oxidative stress-related pathways (Keles et al. 2025) Potential mechanisms include processes reported in the literature involving reactive oxygen species (ROS),(Bektas et al. 2020, 2021), impaired DNA repair processes (Guler et al. 2010; Vafaei et al. 2020), and disruption of nephrogenesis-regulating signaling pathways (Pyrpasopoulou et al. 2004; Odaci et al. 2015). However, the long-term impact of prenatal exposure to high-frequency RFR remains largely unknown.

Autophagy, a key cellular degradation and recycling process, plays a crucial role in kidney homeostasis under both physiological and pathological conditions (Ma et al. 2022). Its dysregulation has been implicated in various renal disorders, including acute kidney injury, chronic kidney disease, and renal aging (Vafaei et al. 2020). Some studies suggest that RFR exposure interferes with autophagic processes, leading to excessive activation or inhibition of autophagy-related pathways (Bertuccio et al. 2023; Jangid et al. 2024; Kirimlioglu et al. 2024; Kartal et al. 2025). While increased autophagic activity may result in excessive degradation of essential cellular components, insufficient autophagy can cause the accumulation of damaged organelles and toxic aggregates, further contributing to renal dysfunction (Arab et al. 2022; Liang et al. 2022). However, the specific impact of high-frequency RFR on renal autophagy remains largely unexplored.

To address this knowledge gap, our study investigates the effects of prenatal 3.5 GHz RFR exposure on renal tissue, focusing on DNA integrity and autophagy-related markers. We hypothesize that high-frequency RFR exposure may be associated with alterations in key cellular homeostasis mechanisms. Given the vulnerability of the developing kidney to environmental stressors, understanding the long-term consequences of prenatal RFR exposure is important. By comparing our findings with previous research on lower-frequency RFR, this study provides additional insights into the biological effects of high-frequency wireless communication technologies on renal development and function. The study includes 3.5 GHz RF exposure groups during the entire pregnancy and the last 2 weeks of pregnancy, highlighting the effects of RF exposure at different gestational periods.

## Materials and methods

### Animal models and classification

Ethical approval for this study was obtained from the Animal Experiments Local Ethics Committee of Istanbul Bagcilar Training and Research Hospital (No. 2024/52). A total of 24 pregnant Wistar Hannover rats (with an average gestation period of 21–22 days) were randomly assigned to three experimental groups (*n* = 8 dams per group): sham control, exposure during the last 2 weeks of gestation (D2T), and exposure throughout gestation (D3T).

A total of 24 male offspring were included in the final analyses (Sham control: *n* = 8; D2T: *n* = 8; D3T: *n* = 8). To avoid pseudoreplication and control for litter effects, the litter was considered the experimental unit, and one randomly selected male offspring from each litter was included in all analyses.

To prevent external radiofrequency interference, all groups were housed in separate Faraday cages throughout the exposure period. After birth, offspring were maintained under standardized environmental conditions (temperature: 22 ± 1 °C, relative humidity: 50 ± 10%, 12-h light/dark cycle) until 6 months of age.

At 6 months of age, animals were anesthetized with ketamine (75 mg/kg) and xylazine (10 mg/kg) and euthanized by intracardiac exsanguination. Kidney tissues were harvested for histopathological, immunohistochemical, and molecular analyses.

### Exposure and field measurements

A signal generator (model 3500 PM10, Everest Comp., Adapazarı, Turkey) producing a 3.5 GHz radiofrequency waveform was used for exposure standardization. The power output of the signal generator was maintained at 1 W throughout the exposure period. The antenna, designed to mimic a conventional mobile phone antenna, was positioned centrally approximately 50 cm above the exposure cage.

Pregnant rats in the experimental groups were exposed to 3.5 GHz radiofrequency radiation (RFR) for 2 h/day during gestation only. Sham control animals were maintained under identical experimental conditions, except that the signal generator remained switched off. All animals were housed in specially designed Plexiglas cages without metallic components to minimize electromagnetic reflection and distortion. Even the water bottle tips were constructed from Plexiglas tubing to preserve field integrity. Animals were allowed to move freely within the cages.

Each experimental group was housed in a separate Faraday cage throughout irradiation to eliminate environmental electromagnetic interference. The signal generator was positioned outside the Faraday enclosure, whereas the antenna extended inside the cage. A metal reflector was positioned above the antenna to improve field uniformity.

After birth, male offspring were separated and were not exposed to any additional RFR until 6 months of age. Therefore, all observed findings reflected prenatal-only exposure.

Electric field measurements were performed using a calibrated EMR-300 probe (NARDA, Pfullingen, Germany). Field strength was measured at five predefined locations within the exposure cage (four corners and center), yielding values of 24, 26, 26.3, 27, and 28 V/m, respectively, indicating relatively homogeneous electromagnetic field distribution across the exposure area (Gelenli Dolanbay et al. 2025).

### Specific absorption rate (SAR) analysis

CST Studio Suite software (CST AG, Darmstadt, Germany) was used to simulate specific absorption rate (SAR) distribution at the rat uterus. The experimental exposure setup was reconstructed in detail to closely replicate the actual irradiation conditions. A high-resolution voxel-based anatomical rat model derived from computed tomography (CT) datasets available in the CST biological model library was used for electromagnetic simulations. The antenna system, reflector, Plexiglas cage structure, and animal positioning were incorporated into the simulation model.

Electromagnetic calculations were performed using the finite integration technique (FIT), enabling simultaneous electromagnetic and thermal simulations. To validate the computational model, simulated electric field values were compared with experimentally measured values obtained using the EMR-300 field probe (NARDA, Pfullingen, Germany). Measurements were obtained from five locations across the exposure plane (four corners and center). No significant variation was observed among measured values, which ranged between 24 and 28 V/m, confirming relatively homogeneous field distribution.

Based on the measured field strengths, peak spatial specific absorption rate (psSAR) values were calculated at 3.5 GHz using CST Studio Suite software and the average measured electric field intensity (Gelenli Dolanbay et al. 2025).

Temperature variations within the exposure environment were continuously monitored using a calibrated type-K thermocouple probe (accuracy ± 0.1 °C) positioned adjacent to the animal exposure area inside the Plexiglas cage. Temperature measurements were recorded at 1-min intervals throughout the entire exposure period using a digital acquisition system. Baseline temperature values were obtained before exposure and continuously compared with measurements recorded during RFR exposure.

No statistically or biologically significant temperature increase was detected during 3.5 GHz exposure at 1 W output power. The maximum observed temperature variation remained within ± 0.2 °C of baseline values, which is within the physiological fluctuation range for laboratory rodents.

All metallic materials were excluded from the exposure environment to minimize electromagnetic reflection artifacts. Thermal effects were additionally considered during SAR simulations; however, neither computational nor experimental analyses demonstrated evidence of thermal overload conditions (Gelenli Dolanbay et al. 2025).

### Tissue processing and immunohistochemical analysis

Kidney tissue specimens fixed in 10% neutral buffered formalin (NBF) were routinely processed, embedded in paraffin blocks, and sectioned at 5 μm thickness for hematoxylin–eosin (H&E) staining. Sections prepared for immunohistochemistry (IHC) were deparaffinized in xylene and rehydrated through graded ethanol series followed by distilled water for 5 min. Subsequently, sections were rinsed with TBS-T buffer (480 mL distilled water, 20 mL Tris-buffered saline [TBS], and 500 µL Tween-20) and incubated for 15 min at 37 °C in 0.1% Triton X-100 prepared in phosphate-buffered saline (PBS). Antigen retrieval was performed using heat-induced epitope retrieval in citrate buffer (pH 6.0) with microwave treatment at 800 W until boiling, followed by 2-min cooling intervals, repeated five times (Magaki et al. 2019).

### Histological examination and evaluation

Histological evaluation was performed under light microscopy (Olympus BX53). Histopathological alterations, including glomerular atrophy, vacuolization (Vac), tubular dilation (TubDil), hemorrhage, and cast formation (Cast), were assessed among the groups (Okatan et al. 2018).

The immunohistochemical (IH) score was determined using a semiquantitative approach, as previously described by Kanbe et al. (2014). The quantification of staining intensity was achieved using a visual analogue scale (VAS) ranging from 0 to 100 mm. The scoring system was categorized into six grades based on the percentage of positive staining: The following gradings are employed: grade 0 (0%), grade 1 (< 5%), grade 2 (5–20%), grade 3 (20–40%), grade 4 (40–60%), and grade 5 (60–100%). The cumulative IH grades obtained from four different microscopic fields were summed to generate the final IH score for each renal specimen. For instance, a total IH score of 11 was yielded by scores of 2, 3, 1, and 5 obtained from four different microscopic fields.

Histological and immunohistochemical evaluations were performed independently by investigators blinded to the experimental groups to minimize observer bias.

### Autophagic activity (immunohistochemistry)

Immunohistochemical staining for Beclin-1 and LC3 was performed to assess autophagy-related protein expression in renal tissue. Following antigen retrieval and blocking procedures, tissue sections were incubated overnight at 4 °C with primary antibodies against Beclin-1 (Lot no: 230103481; Epitomics, Burlingame, CA, USA; dilution 1:200) and LC3 (Lot no: orb33328; Biorbyt, Cambridge, UK; dilution 1:200). On the following day, sections were incubated with horseradish peroxidase (HRP)-conjugated secondary antibodies, and immunoreactivity was visualized using 3,3′-diaminobenzidine (DAB) chromogen (Maroni et al. 2024). Sections were counterstained with hematoxylin and mounted with synthetic mounting medium.

Negative control sections were processed simultaneously by omitting the primary antibody to confirm staining specificity. Antibody specificity was additionally supported by manufacturer validation data and previous studies demonstrating the use of these antibodies in renal tissue. The observed staining pattern was consistent with the expected cytoplasmic localization reported in the literature.

To evaluate interobserver reliability, two independent blinded observers analyzed 24 samples representing different IH score stages for Beclin-1 and LC3 on two separate occasions.

As immunohistochemistry alone does not directly assess autophagic flux, the findings were interpreted as changes in autophagy-related protein expression rather than definitive evidence of altered autophagic activity or impaired autophagic flux.

### DNA damage (comet assay)

DNA damage in kidney tissue was assessed using the neutral comet assay. Briefly, a 0.5 g kidney tissue sample was excised, minced, and suspended in 2 mL cold PBS. The mixture was agitated at 500 rpm for 5 min and subsequently incubated on ice for 10 min. The resulting supernatant was used for comet assay slide preparation according to a standardized protocol (Baran et al. 2022).

Slides were examined using an Olympus BX51 fluorescence microscope (Tokyo, Japan) at 200× magnification. One slide was prepared for each animal, resulting in a total of 24 slides. A total of 50 randomly selected cells from each animal were analyzed using the Comet Assay Software Project (CASP v1.2.2; Windows 2010), an automated image analysis system.

DNA damage parameters including head length, tail length, comet length, head DNA (%), tail DNA (%), tail moment, and olive tail moment were quantified. Cells exhibiting comet-like tail formation were classified as DNA-damaged, whereas nuclei without detectable tails were considered undamaged (Yalcin et al. 2023).

All comet assay analyses were performed in a blinded manner to ensure objective evaluation and reproducibility.

### Statistical analysis

Statistical analyses were performed using SPSS software (Version 20.0; IBM SPSS Inc., Chicago, IL, USA). Data are presented as mean ± standard deviation (SD). The normality of distribution for OTM, TM, TAIL DNA, HEAD DNA, L. COMET, L. TAIL, and L. HEAD variables was assessed using the Kolmogorov–Smirnov test. For variables that did not show a normal distribution (OTM, TM, TAIL DNA, HEAD DNA, L. COMET, and L. TAIL), the Kruskal–Wallis test was applied to compare differences among groups. When significant differences were detected, post hoc pairwise comparisons were performed using the Conover test (Conover 1980). For normally distributed data (L. HEAD), one-way analysis of variance (ANOVA) was used to evaluate group differences, followed by Duncan’s multiple range test for post hoc comparisons. Duncan’s test was selected owing to its suitability for identifying intergroup differences in balanced experimental designs with equal sample sizes.

All statistical tests were two-tailed, and exact *p*-values were reported where applicable. Statistical significance was defined as *p* < 0.05.

## Results

The peak spatial specific absorption rate (psSAR), calculated in accordance with IEEE/IEC 62704-1 standards, was 0.06622 mW/g for 1 g tissue and 0.03825 mW/g for 10 g tissue, indicating low-level prenatal exposure under the experimental conditions.

**Fig. 1:**
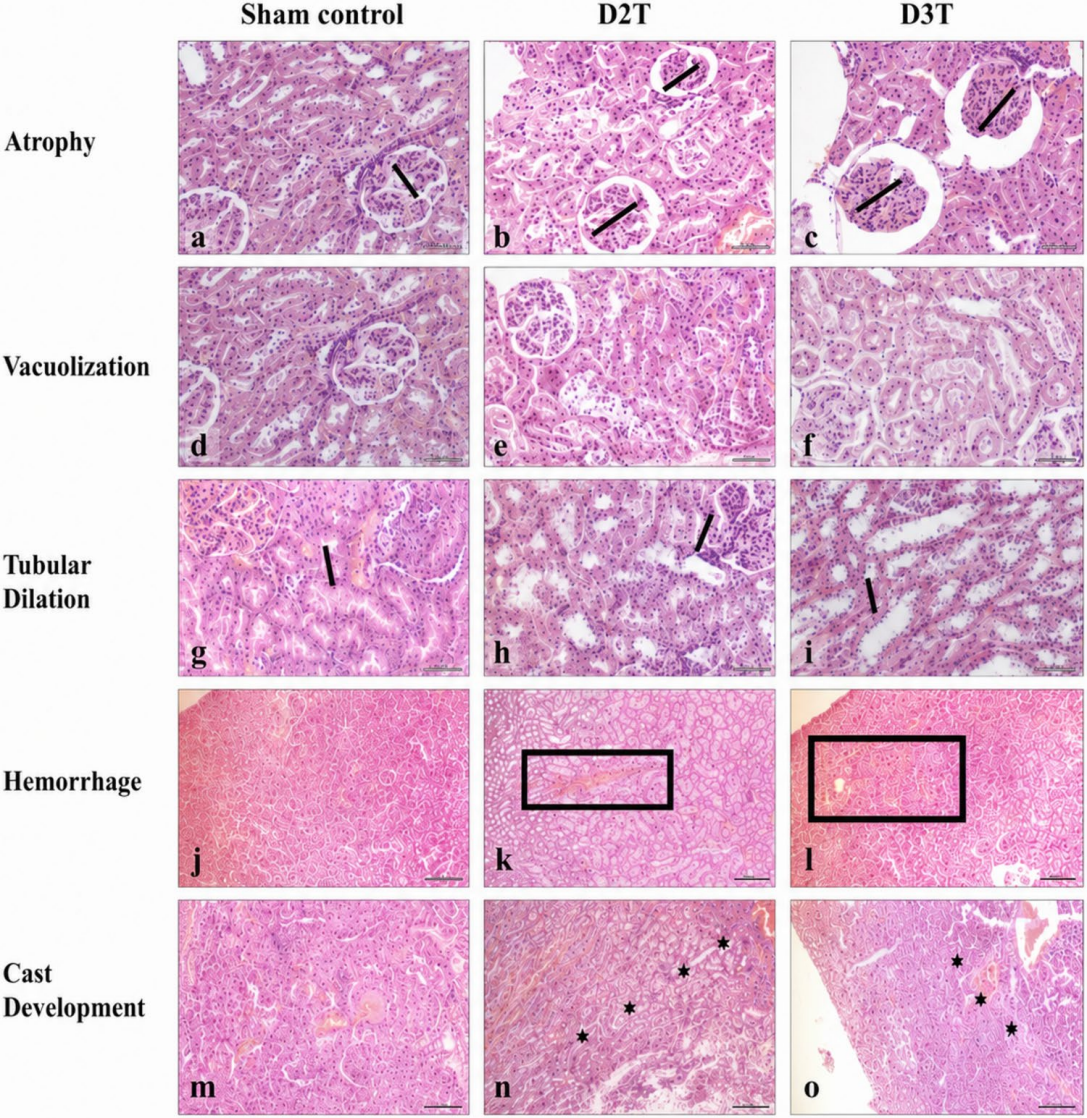
Hematoxylin and eosin (H&E)-stained sections of kidney tissue from radiofrequency radiation (RFR)-exposed rats. Panels **a**, **d**, **g**, **j**, and **m** represent the sham conrol group. Panels **b** and **c** show tubular atrophy; panels **e** and **f** demonstrate cytoplasmic vacuolization; panels **h** and **i** illustrate tubular dilatation; panels **k** and **l** indicate hemorrhage; and panels **n** and **o** reveal cast formation in the D2T and D3T exposure groups, respectively. Scale bar = 50 µm

Histopathological evaluation of renal tissues demonstrated significant intergroup differences in glomerular atrophy, tubular vacuolization, tubular dilatation, cast formation, Beclin-1 expression, and LC3 expression (Table 1, Fig. 1). Hemorrhage did not differ significantly among groups (*p* = 0.41).

**Table 1:** Differences in histopathological parameters between groups

Group	n	Atrophy (*x* ± Sx)	Vac (*x* ± Sx)	TubDil (*x* ± Sx)	Hemorrhage (*x* ± Sx)	Cast (*x* ± Sx)	Beclin-1 (*x* ± Sx)	LC3 (*x* ± Sx)
Sham control	8	2.38 ± 0.32^b^	1.88 ± 0.52^b^	2.09 ± 0.23^b^	3.75 ± 0.75	0.63 ± 0.32^bc^	2.91 ± 0.21^b^	2.63 ± 0.19^b^
D2T	8	4.50 ± 0.85^b^	1.50 ± 0.38^b^	4.59 ± 0.19^a^	5.50 ± 0.76	0.50 ± 0.19^c^	3.84 ± 0.19^a^	4.03 ± 0.17^a^
D3T	8	6.88 ± 0.91^a^	4.00 ± 0.50^a^	5.63 ± 0.27^a^	5.50 ± 1.20	1.63 ± 0.38^ab^	4.00 ± 0.19^a^	4.19 ± 0.15^a^
*p*		**	**	***	NS	*	***	***
*p* value		**0.0021**	**0.0026**	**1.42 × 10**^**⁻17**^	**0.41**	**0.0155**	**2.22 × 10**^**⁻4**^	**2.54 × 10**^**⁻9**^

a,b,c Different superscript letters indicate significant differences between groups, Vac tubular vacuolization, *TubDil* tubular dilatation, *Cast* cast formation. *p < 0,05, **p < 0,01, ***p < 0,001, *NS* not significant. *x* mean, *Sx* SEM

Bold values indicate statistically significant differences compared with the sham group

Glomerular atrophy was significantly increased in the D3T group compared with both the sham control and D2T groups (*p* = 0.0021), indicating a gestation-duration–dependent effect of prenatal RFR exposure (Fig. 1c).

Tubular vacuolization showed a significant increase in the D3T group compared with both the sham control and D2T groups (*p* = 0.0026), whereas the D2T group showed intermediate values relative to controls (Fig. 1d–f).

Tubular dilatation was significantly elevated in both exposure groups compared with the sham control group, with the highest levels observed in the D3T group (*p* = 1.42 × 10^−17^) (Fig. 1g–i).

Cast formation showed a significant intergroup difference (*p* = 0.0155), with higher values observed in the D3T group compared with the other groups (Fig. 1m–o).

**Fig. 2:**
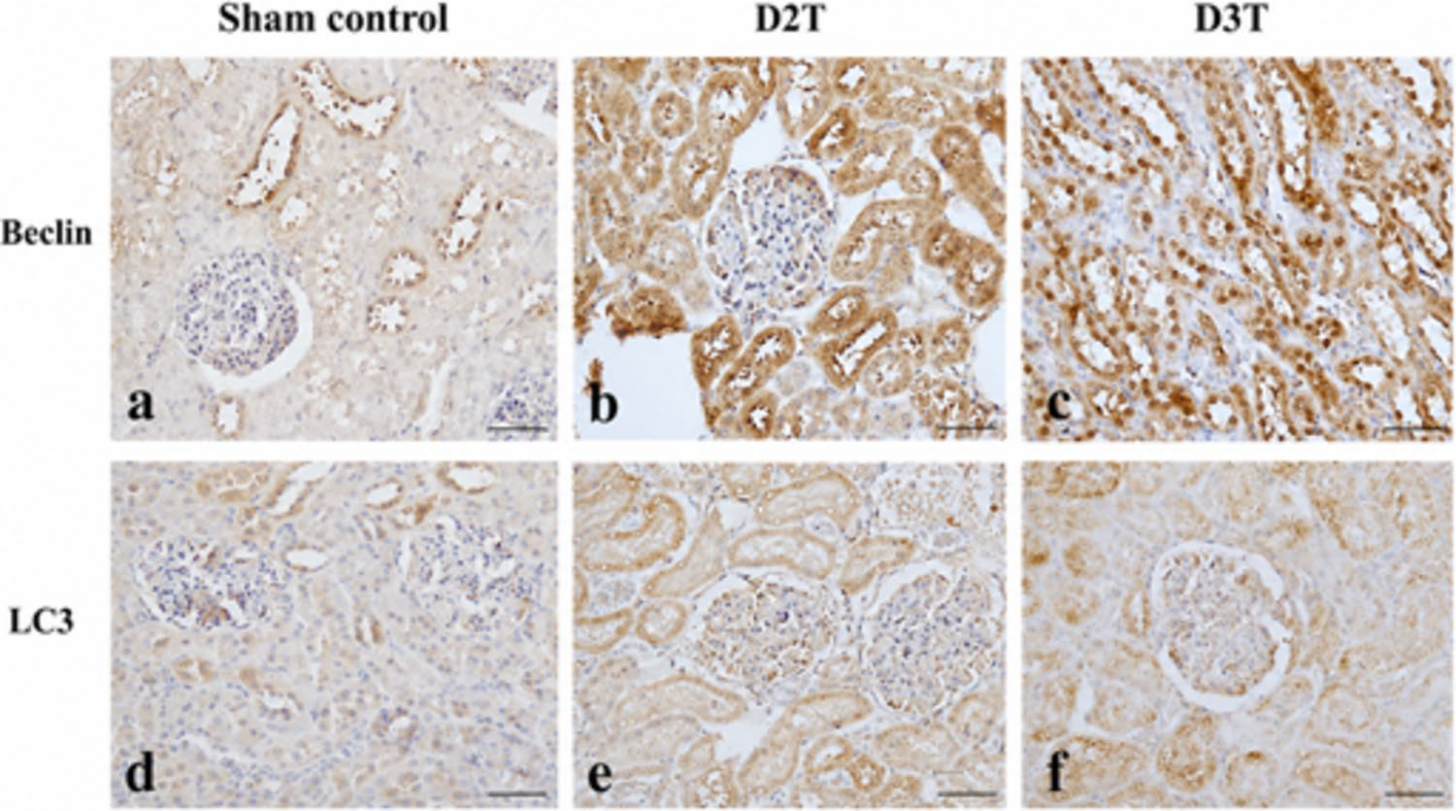
Immunohistochemical staining of kidney tissue from radiofrequency radiation (RFR)-exposed rats. Panels **a** and **d** represent the sham control group. Panels **b** and **c** show increased Beclin-1 expression, whereas panels **e** and **f** demonstrate elevated LC3 expression in the D2T and D3T groups, respectively. Positive immunoreactivity was observed predominantly in proximal tubular epithelial cells. Scale bar = 200 µm for panel d; 50 µm for panels a, b, c, e, and f

Beclin-1 expression was significantly increased in both D2T and D3T groups compared with sham control (*p* = 2.22 × 10^−4^), with maximal expression in the D3T group (Fig. 2a–c).

Similarly, LC3 expression was significantly elevated in both exposure groups compared with controls (*p* = 2.54 × 10^−9^), again showing the highest levels in the D3T group (Fig. 2d–f).

**Fig. 3:**
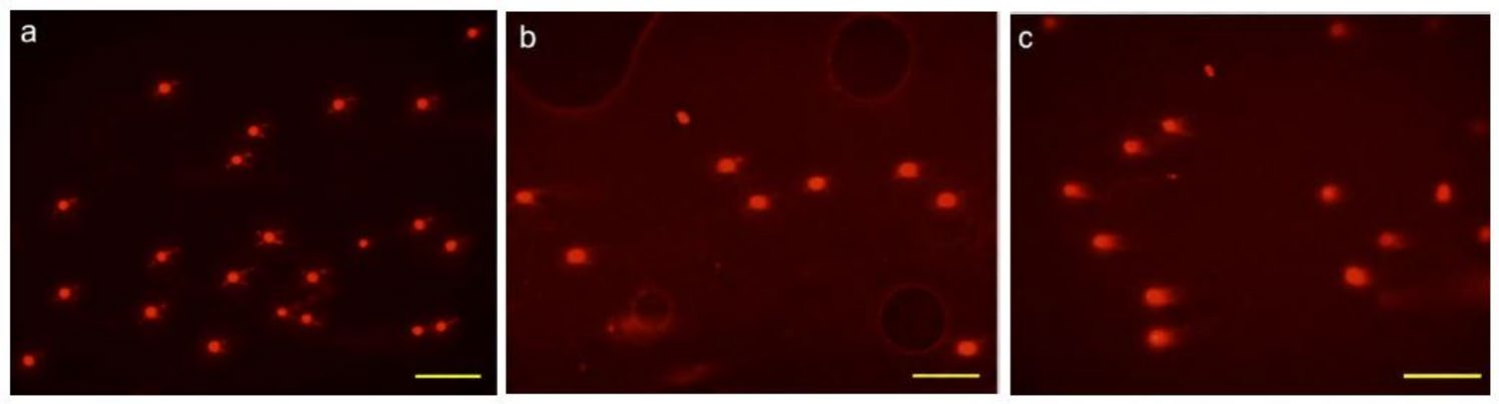
Representative images of the neutral comet assay performed in kidney cells. Panel **a**: sham control group (Tail DNA: 4.06%); panel **b**: D2T group (Tail DNA: 19.92%); panel **c**: D3T group (Tail DNA: 25.74%). Ethidium bromide staining, original magnification × 200. Scale bar = 200 µm

DNA damage analysis demonstrated significant intergroup differences across all comet assay parameters, including head length, tail length, total comet length, head DNA percentage, tail DNA percentage, tail moment (TM), and olive tail moment (OTM) (Table 2, Fig. 3; all *p* values as reported in Table 2).

**Table 2:** Differences in DNA damage parameters between groups

Group	*n*	L.Head (*x* ± Sx)	L.Tail (*x* ± Sx)	L.Comet (*x* ± Sx)	Head DNA (*x* ± Sx)	Tail DNA (*x* ± Sx)	TM (*x* ± Sx)	OTM (*x* ± Sx)
Sham control	8	129 ± 2.76^c^	24.8 ± 1.34^b^	153.8 ± 3.62^b^	95.94 ± 0.13^a^	4.06 ± 0.13^c^	1.22 ± 0.06^c^	2.16 ± 0.12^c^
D2T	8	227.24 ± 5.96^a^	175.02 ± 6.54^a^	408.02 ± 9.42^a^	80.08 ± 0.65^b^	19.92 ± 0.65^b^	35.96 ± 2.23^b^	29.08 ± 1.34^b^
D3T	8	208.24 ± 4.13^b^	172.1 ± 6.93^a^	377.44 ± 9.48^a^	74.26 ± 0.27^c^	25.74 ± 0.27^a^	44.52 ± 2.06^a^	33.54 ± 0.96^a^
*p*		***	***	***	***	***	***	***
*p*-value		3.3725 × 10^−17^	7.9384 × 10^−4^	1.3605 × 10^−22^	2.0441 × 10^−17^	1.1686 × 10^−17^	1.2233 × 10^−10^	4.2037 × 10^−12^

a,b,c Different superscript letters indicate significant differences between groups. *L*. length, *TM* tail moment, *OTM* olive tail moment. ***p < 0,001, *x* mean, *Sx* SEM

Bold values indicate statistically significant differences compared with the sham group

Both D2T and D3T groups showed significant increases in DNA damage indices compared with the sham control group (all *p* < 0.001 in Table 2), with more pronounced alterations in the D3T group, indicating a duration-dependent genotoxic effect of prenatal 3.5 GHz RFR exposure.

## Discussion

The increasing deployment of wireless communication systems operating in the 3.5 GHz frequency range has raised ongoing scientific interest regarding potential biological effects of radiofrequency radiation (RFR), particularly during early developmental periods. In the present experimental study, prenatal exposure to 3.5 GHz RFR was associated with structural and molecular alterations in rat kidney tissue at 6 months of age, including histological changes, increased DNA damage indices, and modulation of autophagy-related protein expression. While these findings do not establish causality in humans, they contribute to the expanding experimental literature evaluating frequency-specific biological responses to RFR exposure.

Histological evaluation demonstrated glomerular atrophy, tubular dilation, vacuolization, and cast formation in RFR-exposed groups, with more pronounced changes in the D3T group. These findings are consistent with previous experimental reports at lower frequencies (900 MHz–2.45 GHz), suggesting that renal tissue may exhibit sensitivity to RF-related stress across different exposure ranges (Oktem et al. 2005; Ozorak et al. 2013; Ulubay et al. 2015). The graded morphological alterations observed between exposure groups may indicate a relationship between duration of prenatal exposure and tissue susceptibility. However, differences in experimental design and dosimetry across studies limit direct comparisons.

In the present study, increased Beclin-1 and LC3 immunoreactivity was observed in renal tissue following prenatal RFR exposure. These proteins are widely used markers associated with autophagy-related pathways. However, since autophagic flux was not directly assessed, these findings should be interpreted as changes in autophagy-related protein expression rather than definitive evidence of altered autophagic activity. This interpretation is consistent with previous studies reporting variable autophagy responses following RF exposure, depending on frequency, exposure duration, and tissue-specific factors (Kim et al. 2016; Sannino et al. 2022; Golbach et al. 2015).

Comet assay analysis demonstrated significant increases in DNA fragmentation parameters in RFR-exposed groups, indicating increased genomic stress in renal tissue. These findings are in agreement with previous experimental studies reporting DNA damage and strand breaks following RF exposure, which have been discussed in the literature in the context of oxidative stress-related processes (Dasdag and Akdag 2016; Alkis et al. 2019). Nevertheless, inconsistencies in the literature suggest that genotoxic outcomes may be highly dependent on exposure conditions, biological context, and methodological differences.

The developing kidney appears to be particularly sensitive to environmental stressors, including prenatal RF exposure. Experimental developmental studies have reported structural renal alterations following embryonic exposure, including tubular and glomerular abnormalities (Rehman 2015; Dsilva et al. 2022). These findings, together with maternal exposure models, suggest that prenatal life may represent a critical window of susceptibility for renal tissue development (Raouf and Girgis 2022; Celegen et al. 2024).

Epidemiological and experimental studies have suggested that prolonged RFR exposure may be associated with cellular stress responses, including processes reported in the literature such as oxidative stress, inflammation, and cellular damage in renal tissues (Zhang et al. 2023; Mahmoudi et al. 2018; Hasan et al. 2021). Therefore, extrapolation of experimental findings to clinical outcomes should be made cautiously.

Several limitations should be acknowledged. Only male offspring were included, which limits sex-based interpretation. In addition, a single frequency (3.5 GHz) and exposure protocol were used, limiting generalizability across different RF conditions. Furthermore, mechanistic interpretation is based on marker expression and DNA damage assessment rather than functional pathway analysis or interventional validation.

Future studies should incorporate oxidative stress biomarkers, autophagic flux measurements, and senescence-related end points to better elucidate underlying mechanisms. Comparative studies across different frequencies and exposure intensities would also help clarify dose–response relationships and biological thresholds.

Prenatal exposure to 3.5 GHz RFR was associated with renal histological alterations, increased DNA damage indices, and changes in autophagy-related protein expression in adult rat offspring. These findings extend previous experimental observations at lower frequencies and suggest that the developing kidney may exhibit increased susceptibility to prenatal RF exposure under controlled experimental conditions. However, given the limitations of the study design, these results should be interpreted as experimental evidence of biological association rather than causal evidence of disease or functional impairment in humans.

## Data Availability

The data that supports the findings of this study are available from the corresponding author upon reasonable request.

## Declarations

### Use of generative AI assisted tools

The manuscript was reviewed for language clarity and readability using AI-assisted language editing tools. The authors take full responsibility for the content of the manuscript.
